# “Showing some care”: interest-holders’ perspectives on addressing health-related social conditions in ovarian cancer

**DOI:** 10.1093/oncolo/oyag077

**Published:** 2026-03-16

**Authors:** Anand R Habib, Sarah Pitafi, Elena Ratner, Emily Abramsohn, Stacy Tessler Lindau, Cary P Gross, Dena Schulman-Green

**Affiliations:** Division of Hospital Medicine, University of California San Francisco, San Francisco, CA 94143, United States; National Clinician Scholars Program, Yale School of Medicine, New Haven, CT 06510, United States; Harvard Medical School, Boston, MA 02115, United States; Department of Obstetrics, Gynecology and Reproductive Sciences, Yale School of Medicine, New Haven, CT 60637, United States; Department of Obstetrics and Gynecology, University of Chicago, Chicago, IL 60637, United States; Department of Obstetrics and Gynecology, University of Chicago, Chicago, IL 60637, United States; Section of Geriatrics and Palliative Medicine, Department of Medicine, University of Chicago, Chicago, IL 60637, United States; Comprehensive Cancer Center, University of Chicago, Chicago, IL 60637, United States; National Clinician Scholars Program, Yale School of Medicine, New Haven, CT 06510, United States; Yale University School of Medicine, New Haven, CT 06510, United States; New York University Rory Meyers College of Nursing, New York, NY 10010, United States

**Keywords:** ovarian cancer, social conditions, health equity

## Abstract

**Background:**

Health-related social conditions (HRSCs) are modifiable factors affecting ovarian cancer outcomes. How best to manage HRSCs as part of ovarian cancer care remains unclear. We sought interest-holder perspectives on HRSC assessment and assistance to inform integration of social care with gynecologic oncology practice.

**Methods:**

In this qualitative descriptive study, we conducted individual, semi-structured interviews with patients with ovarian cancer, their caregivers, and oncology clinicians from an academic medical center. We solicited thoughts on HRSC assessment and assistance, including whether and how HRSC discussions should occur, as well as opinions on a 20-item HRSC assessment and a community resource referral document. We analyzed data using the Rigorous and Accelerated Data Reduction (RADaR) technique.

**Results:**

Participants (*n* = 14) included 5 patients with ovarian cancer, 4 caregivers, and 5 clinicians. Patients and caregivers were open to HRSC conversations with clinicians from various disciplines if undertaken with empathy, respect, and active listening. In addition to common HRSCs like food insecurity and housing instability, participants endorsed attending to religion/spirituality and assessing for financial hardship, interpersonal violence, psychological support, and neighborhood safety. Patients and clinicians characterized the HRSC screening questions as straightforward and in-depth. All participants felt the community resource referral document was well-organized and helpful.

**Conclusion:**

Even in the context of a life-threatening disease, participants affected by and caring for patients with ovarian cancer regard social conditions as relevant to cancer care. Our findings inform practical considerations of who, what, when, where, why, and how HRSC assessment and assistance can be undertaken in oncology.

## Graphical abstract

**Graphical Abstract:**
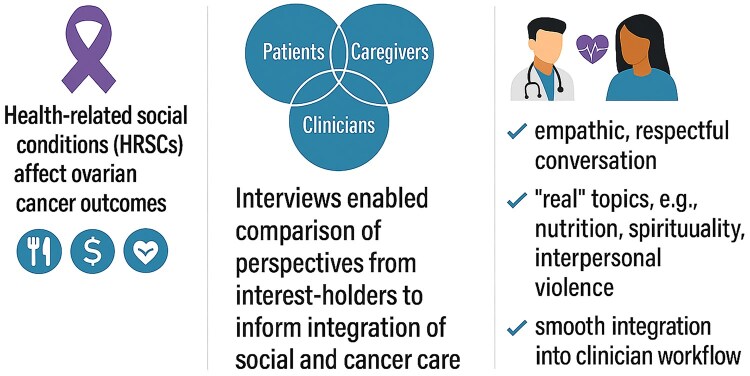

Implications for Practice Findings suggest design of HRSC assessment for people with ovarian cancer should consider relational and longitudinal elements, including the possibility of delivery after rapport is established and reassessing over time as needs change. Practices should consider both clinicians’ desire for EMR documentation and a range of patient preferences for confidentiality. Reliance on self-administered screening may be convenient but also could compromise empathy and confidence for some patients; multi-modal approaches with the option of person-to-person engagement may be optimal. Importantly, screening without assistance can erode trust—patients value knowing help is available even if not immediately used. Universal and routine provision of community resource information offers a cost-effective, stigma-reducing strategy, while community navigators remain essential to support follow-through and reduce patient burden.

## Introduction

Despite advances in cancer prevention, diagnosis, and treatment, barriers to high-quality, equitable cancer care persist.^1–4^ For women with ovarian cancer, the sixth leading cause of cancer-related deaths among women, such barriers produce racial and socioeconomic disparities in treatment access and outcomes.^5–7^ African American women and women of lower socioeconomic status experience delayed diagnosis, less guideline-concordant cancer-directed therapy, and increased cancer-related mortality.^7–9^ Women with ovarian cancer are also vulnerable to financial toxicity, as treatments tend to be expensive, multimodal, and potentially have detrimental effects on health and functioning.^10^^,^^11^

Professional societies recognize that achieving equity in cancer care involves improving access to guideline-concordant care and addressing patients’ health-related social conditions (HRSCs) as “experiences that affect an individual’s health and well-being,” such as food insecurity and housing instability.^12–15^ The American Society of Clinical Oncology (ASCO) and Society of Gynecologic Oncology policy statements build upon a 2019 National Academies report that called for greater awareness of HRSCs and offered a framework for integration of social and clinical care through increased screening and documentation, assistance with unmet HRSCs, and adjusting oncologic care to overcome barriers encountered by socioeconomically diverse patients.^14–16^

Yet knowledge gaps remain in how to integrate social care into cancer care. There is no consensus regarding which HRSCs are relevant to oncology, the acceptability of HRSC screening tools, who should screen for and discuss identified needs, and interest-holders’ perspectives on how to respond to unmet HRSCs. Accordingly, we sought perspectives of interest-holders on the integration of social care with gynecologic oncology care to inform integrative efforts.

## Methods

### Study design

In this qualitative descriptive study, we conducted semi-structured, individual interviews with interest-holders between March 2021 and October 2022.^17^ The purpose of interest-holder interviews is to obtain project-relevant information from individuals vested in the success of the work and to elicit their reactions and suggestions regarding poorly understood phenomena.^18^ Qualitative descriptive studies support small, purposively selected samples to obtain in-depth, project-relevant perspectives.^19^^,^^20^ The Yale Institutional Review Board approved the study. Reporting was guided by the Standards for Reporting Qualitative Research (SRQR).^21^

### Sample

We purposively sampled and enrolled women with ovarian cancer, their caregivers, and oncology clinicians at an urban, tertiary-level academic medical center as interest-holders given their involvement in clinic-based discussions of HRSCs in the context of ovarian cancer care. Eligible patients were English- and/or Spanish-speaking, aged 18+ years, 1-6 months after initial diagnosis of or post-recurrence of stage III/IV epithelial ovarian cancer and receiving or planning to initiate treatment within 2 weeks of our contact. Eligible caregivers were aged 18+ years and of any relationship (eg, partner, child, friend) to a patient with ovarian cancer. Patients with known brain metastases or cognitive impairment and their associated caregivers were excluded. Oncology clinicians were eligible if they cared for patients with ovarian cancer at the participating site.

### Procedures

After identifying potential patients through the electronic medical record (EMR), we asked treating clinicians to introduce the study to them, and we approached interested patients. All participants received a written study information sheet and provided verbal consent. We identified caregivers through patient nomination.

Interview questions (Table 1) were informed by the Consolidated Framework for Implementation Science,^22^ an implementation determinants framework, and covered: (1) comfort in discussing HRSCs in a clinical setting and the scope of relevant HRSCs; (2) opinions on a 20-item HRSC screening instrument (Supplemental File S1**)** that included validated questions from the Accountable Health Communities tool,^23^ the Medical Expenditure Panel Survey,^24^ and the Comprehensive Score for Financial Toxicity—Functional Assessment of Chronic Illness Therapy instrument^25^; and (3) feedback on the design and content of a community resource referral document that provided information on community-based organizations addressing HRSCs (Supplemental File S2).

**Table 1.** Interview questions for patients, caregivers, and clinicians.

Participant group(s)	Interview questions
**For patients and caregivers**	Do you think that any needs are missing from this picture (Figure 1A)? How would you feel about being asked about your needs in these areas in this clinic? Which health professional would you feel comfortable discussing these needs with? Is there anything in particular that you liked or disliked about the survey instrument that you’d like to share? Did the survey instrument feel burdensome? What changes would you suggest to (the community resource referral document)? What did you like most about (the community resource referral document)?
**For clinicians**	How would you feel about your patients being asked regularly about their social and economic needs, such as housing and food insecurity, as part of their clinic visits? How frequently do you think your patients should be asked about their social and economic needs? Would you want to be able to view your patients’ social and economic needs in their health records? Why or why not? Is there anything in particular that you liked or disliked about the survey instrument that you’d like to share? Did the survey instrument feel burdensome? What changes would you suggest to (the community resource referral document)? What did you like most about (the community resource referral document)?

**Figure 1.**
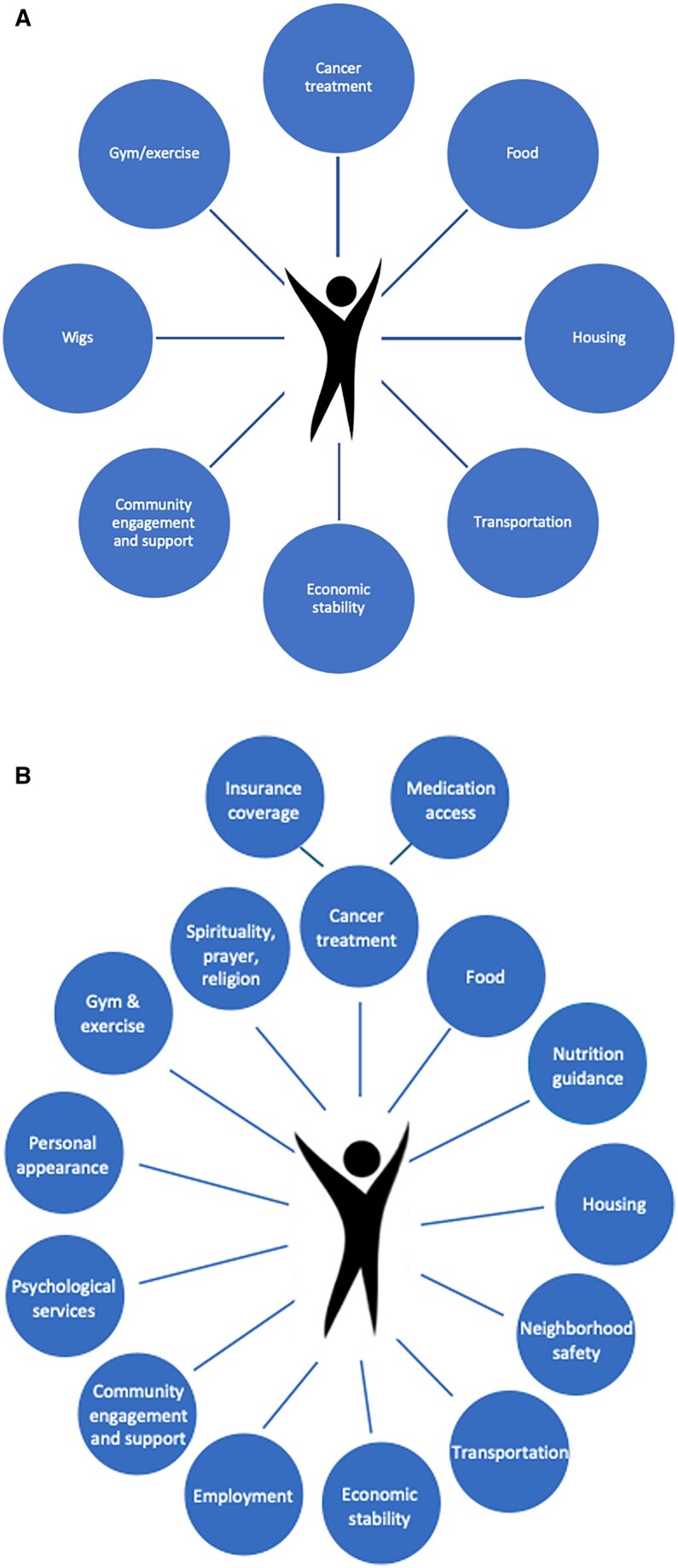
Conceptual diagram outlining health-related social conditions (HRSCs) in ovarian cancer—(A) original diagram (B) revised diagram.

The community resource referral document was developed using a patient-centered design used in other disease settings; its acceptability among women with ovarian cancer has not been assessed.^26^ Although the community resource referral document would, in practice, be personalized and auto-generated, for this study, we sought participants’ feedback on a standardized version, generated from a live platform that provides regularly updated information. To ground discussions of what might constitute an HRSC, we used a conceptual diagram (Figure 1A). Among oncology clinicians, we additionally sought thoughts on viewing patients’ HRSC information within the EMR and on how frequently HRSC discussions should occur.

Two post-undergraduate research assistants with no prior relationship with participants conducted interviews. Both research assistants had prior training and experience in qualitative research. Study-specific training was provided to ensure familiarity with the interview guide and study procedures and to prepare them to conduct interviews with historically marginalized populations and address sensitive topics, including strategies for building rapport, responding to participant distress, and maintaining ethical and respectful engagement throughout data collection. Interviews were conducted via a HIPAA-compliant videoconference platform that permitted audio-recording and transcription. Interview transcripts were stored locally on password-protected computers and only accessible to study team members. All participants received a $25 gift card.

### Data analysis

We used the Rigorous and Accelerated Data Reduction (RADaR) method to organize, reduce, code, and analyze data.^27^ RADaR comprises a 5-phase method to expedite analysis of actionable, targeted qualitative data.^28^ Four coders worked in teams of 2 to analyze data from each participant group separately.

Starting with patient data, coders first formatted all transcripts uniformly and familiarized themselves with the data. In Phase 2, coders organized data into an “all-inclusive” table that included column headings for transcript number, research question, participant response, codes, and notes.^28^ Next, coders collaboratively reduced the data, only leaving concepts that directly addressed each interview question. As our interview questions were quite targeted, multiple rounds of data reduction were not required. Notably, this process of data reduction could yield as an output less detailed and rich data than might be expected from other qualitative approaches given the intent to focus only on interview responses directly connected to the questions posed to interviewees. In Phase 4, coders worked independently and then synchronously to identify codes and develop a code key capturing concepts of interest, periodically discussing it with the full study team. Coders applied the final code key to all data. These procedures were repeated with the caregiver and clinician data; coders used the patient code key as an *a priori* code key for the caregiver data, as the interview questions were identical but worded for caregivers’ perspectives. Finally, coders discussed interpretations together and with the full team.

### Trustworthiness and rigor

We strengthened the credibility of our findings by using a semi-structured interview guide, probing for depth during interviews, and engaging in iterative, team-based data review. We supported dependability by carefully documenting each step of our analytic process and using structured data reduction matrices. To further enhance trustworthiness, coders maintained a running memo of coding and analytic decisions for each dataset. We supported confirmability by grounding interpretations in participant data and engaging in team discussions to minimize individual bias. Finally, we addressed transferability by providing a detailed description of the study context and participant characteristics, enabling assessment of the applicability of our findings to other settings.

## Results

Interviews (*n* = 14; 5 patients, 4 caregivers, 5 clinicians) lasted about 30 minutes each. The patient/caregiver code key comprised 5 codes with 26 subcodes. The clinician code key comprised 6 codes with 21 subcodes (Supplemental Files S3 and S4). Tables 2-4 present complete data. Below, we highlight findings from questions asked of only one participant group as well as findings that were unique to a particular participant group for those questions posed to multiple groups.

**Table 2.** Patient, caregiver, and clinician perspectives on health-related social conditions (HRSCs), including salient codes.

Interview topic	Salient codes from patients with ovarian cancer (*n* = 5)	Exemplary patient quotes	Salient codes from caregivers (*n* = 4)	Exemplary caregiver quotes	Salient codes from clinicians (*n* = 5)	Exemplary clinician quotes
**Scope of HRSCs affecting woman with ovarian cancer**	Spirituality and prayer* Psychological services* Employment*	Spirituality/prayer: *“What about spiritual… feeling the need to be connected to a place of worship, or a meditation class, or a yoga class or something that is more than just gym and exercise…?*” Psychological services: *“What about community engagement and support? Does that mean psychological services like having a counselor to talk to? Is that part of community engagement and support? I would think that psychological services would be important.”* Employment: *“…my diagnosis and [the] pandemic announcement all came together at… the same time… [it] made some real difficulties for thinking about my employment.”*	Spirituality and prayer* Psychological services* Employment* Nutrition guidance	Spirituality/prayer: *“I can ask if you could include… prayer because my sister leans a lot on God more than she relies on me! It’s a big part of her life”* Psychological services: *“I’d also throw in… mental health… I see the depression and sadness that surrounds cancer and I think it is extremely lonely if you have to go through this without family around.”* Employment & Nutrition guidance: *“…specific guidance on diet is important too on managing the symptoms of cancer. I feel like with my mother I have seen her go through so much and she would benefit from that support so I think it should be named clearly…Employment too.”*	Religion Medication access Insurance coverage	Religion: *“[S]ome people really value religion. I don’t know if that would fall into community engagement and support.”* Medication access & Insurance coverage: *“[M]edication access is a huge concern, and insurance for cancer patients…That’s something that should be on that wheel more clearly…because really you’re asking the financial questions.”*
**HRSC discussions in the clinical setting**	Yes, ask (2) Ambivalent (1) No response (2)	Yes: “*I’m an open person, I’m willing to talk about anything.”* Ambivalent: *“If I was understanding that the context in which they were being asked… so that if needs in any of these areas were uncovered that I could be made aware of other resources that could potentially assist me in that, I would have no problem with it.”*	—	—	Yes, ask (3) Ambivalent (2) No, do not ask (0)	Yes: *“I don’t think we do a good job of it… across the nation, especially as providers, but… it’s super important, because sometimes patients have difficulty or feel a sense of embarrassment to share those kinds of details, which could be a barrier to their care.”* Ambivalent: *“It is a great idea for patients to be asked about their social economic needs, but… the most important thing is that we do not bombard our patients with too many questions about their situation because we will not want them to, really shy away.”*
**Clinicians with whom you are comfortable discussing HRSCs**	Nurse* Social worker* Oncologist* Surgeon Resource counselor Healthcare professional who has had Stage III/IV ovarian cancer Designated point person Community advocate/liaison (eg, religious leader)	Nurse or social worker or resource counselor: *“Honestly, it depends on the person. It could be a counselor, or a social worker, it could be a nurse… It’s got to be the right person, though. They have to be a listener, they have to be empathetic, but at the same time, they can’t be patronizing.”* Surgeon or Oncologist: *“I think it’s been most clearly related to my surgeon and oncologist.”*	Nurse* Social worker* Oncologist* Primary care physician	Social Worker: *“I would feel comfortable speaking to a social worker. Or someone who… recognize[s] that it is not just what is making me or her ill in the moment but how the things around us in our lives can exacerbate so many different things.”* Primary care physician or oncologist: “*[I]f we [are] talking about specifically who we have felt most comfortable with… I would say primary care physician or oncologist since we see them the most now.”*	—	—

**Key**: *Denotes common codes across ≥2 interest holder groups.

**Table 3.** Patient, caregiver, and clinician feedback on the HRSC screening instruments, including salient codes.

Interview topic	Salient codes from patients with ovarian cancer (*n* = 5)	Exemplary patient quotes	Salient codes from caregivers (*n* = 4)	Exemplary caregiver quotes	Salient codes from clinicians (*n* = 5)	Exemplary clinician quotes
**Positive and constructive feedback on the HRSC screening instruments**	**What they liked:** Addresses “real” issues Questions about: Interpersonal violence* Living situation Food situation Transportation **Add questions on:** Nutrition/supplements* Neighborhood safety Prognosis Spiritual issues Health insurance & employment Caregiving responsibilities Rural health issues	Real issues: *“These are good, good, solid, specific questions and things that people in the real world have to think about.”* Living situation: *“[L]iving situation today is a great question. You have a steady place to live today but I’m worried about losing it in the future. That’s so many of us… in particular seniors.”* Nutrition/supplements: *“I don’t know who to trust when it comes to nutrition… they basically serve you coke and potato chips when you go in for chemo, how can I trust them to counsel me about nutrition?”* Neighborhood safety: *“I’m in a metropolitan area, but [also] for patients in rural areas, you have left out how a person feels about the safety of their neighborhood.”*	**What they liked:** Questions about: Interpersonal violence* Finances* **Add questions on:** Nutrition/supplements* Housing details (eg, housing stability, rent/own, mortgage) Mental health support	Interpersonal violence: *“I like that there is a question about abuse… That’s a big issue, and one that I have seen affect family members…and many people…It is sad but important…”* Finances: *“Asking the questions about finances is so important…cancer will make you bankrupt!”* Nutrition/supplements: *“[I]t is a good survey but I would make changes like including questions about nutrition and supplements… There’s a lot of things that I have to keep track of…and help with that…”* Mental health support: *I would also add questions about mental health support specifically too. My mom is lucky that she has me and her other children to look out for her but what if she didn’t?”*	**What they liked:** Items get at important information* Questions about: Interpersonal violence* Financial needs* Family and friends **Add questions on:** Change in housing Hoarding Questions about stairs Places to get furniture Prescription assistance Short & long-term disability Insurance (eg, Social Security, Coverage) House cleaning Senior center programming	Interpersonal violence: *“I realized I am not… talking to patients about… whether they feel safe at home…you are asking [about] domestic violence questions in the middle of the survey. I like that because we never know what a person may or may not be struggling with.”* Finances: *“[T]he financial aspect is huge, because we see that a lot in our ovarian cancers that are treated much like a chronic disease…Lots of times people have high co-pays [for] immunotherapy and some of our oral drugs that we do try to get financial assistance for them. But it’s scary when your doctor saying you need this pill. But you have a $2,000 copay a month for it.”* Hoarding: *“Under the housing section, you don’t screen for hoarding… which really impact[s] discharge and access to visiting nurses and things.”*

**Key:** *Denotes common codes across ≥2 interest-holder groups.

**Table 4.** Patient, caregiver, and clinician feedback on the sample community resource referral document.

Positive and constructive feedback on content and design/approach			Patients (*n* = 5)	Caregivers (*n* = 4)	Clinicians (*n* = 5)
**Liked**	Content	Information all in one place	**✓**	**✓**	
		Holistic & comprehensive	**✓**		**✓**
		Meets a need & focuses on key stressors	**✓**		**✓**
		Personalized to patients’ needs		**✓**	**✓**
		Presence of a primary contact person	**✓**		**✓**
		Offers an alternative to existing supports	**✓**		
		Worth the effort	**✓**		
		Important information (eg, websites, phone numbers)			**✓**
		Includes COVID content			**✓**
		Co-use for patients and clinicians			**✓**
	Design/Approach	Clarity & well-written	**✓**		**✓**
		Color-coded	**✓**	**✓**	
		Layout			**✓**
		Easy to follow			**✓**
		Quick to access			**✓**
		Well-organized	**✓**		
**Disliked**		Burdensome and/or too overwhelmed to use by themselves	**✓**	**✓**	
**Suggestions**	Content	Clarify any out-of-pocket costs for services		**✓**	
		Add new organizations			**✓**
		More examples of resources			**✓**
		Clearer/specific contact information	**✓**		
	Design/Approach	Availability & nature of this tool should be publicized	**✓**		
		Recruit community advocates (eg, church leader)	**✓**		
		Make more concise and/or offer as a table		**✓**	
		People may need a push to use this tool	**✓**		

### Scope of HRSCs

In contemplating the range of HRSCs that might affect women receiving treatment for ovarian cancer and in reviewing the conceptual diagram (Figure 1A), patients and caregivers both suggested adding spirituality and prayer, psychological services, and employment. Caregivers also recommended considering nutritional guidance. Oncologists emphasized patients’ religion and healthcare-associated challenges like medication access and insurance coverage. Caregivers appreciated reflection on HRSCs affecting their loved ones during cancer care: “My mom’s journey…has been challenging ever since her diagnosis. It is really hard to manage her care and the support that she needs to have beyond me at home…so I like the way the diagram puts it all together.” Table 2 summarizes HRSCs considered relevant. Figure 1B provides a revised conceptual diagram based on participants’ responses.

### HRSC discussions

Patients and clinicians were open to discussing HRSCs. Those who expressed ambivalence cautioned that balance (eg, time spent on these discussions versus clinical discussions and breadth and depth of HRSC discussions at each clinical encounter) and context (eg, why discussing HRSCs is relevant to ovarian cancer care) matter given the potentially sensitive topics broached. One clinician felt “it is a great idea for patients to be asked about their social economic needs” but that oncology teams should not “bombard our patients with too many questions about their situation.” A patient emphasized that context matters; she would be willing to discuss HRSCs if she knew she would receive help.

Patients and caregivers reported that they would feel comfortable discussing HRSCs with nurses, social workers, and oncologists. Caregivers noted that patients’ preferences for which clinician took precedence. A caregiver emphasized that the initiator of HRSC discussions had to be “down to earth and friendly and give…the time of day and respect.”

Clinicians generally agreed that viewing patients’ responses to HRSC screening would be helpful: “I want us…to be best equipped to help our patients even if it is not immediately something that I can resolve.” Another clinician added that insight into a patient’s HRSCs would “make you a little bit more sensitive if you know there’s reluctancy to do a certain treatment.” Clinicians underscored the importance of minimizing workflow disruptions: “The medical record system is honestly burdensome as is.” Another clinician endorsed the utility of auto-populating HRSC information into documentation.

Clinicians’ opinions varied regarding appropriate cadence for HRSC discussions. Some suggested broaching the topic at the first clinic visit and quarterly thereafter; others suggested aligning discussions with treatment milestones (eg, initial chemotherapy infusion, remission, maintenance therapy). Clinicians reiterated “balance[e] in how often [they] ask about these deeply personal needs” but stressed that knowing if a patient’s “financial situation changed in any way that affects [their] ability to take care of [their] health” would be helpful.

### HRSC screening instrument

For the HRSC screening instrument (Table 3**)**, participants from all groups appreciated that it addressed “real” topics like interpersonal violence. Caregivers and clinicians found the questions about financial needs pertinent. Participants generally agreed that the instrument was not burdensome. Patients and clinicians characterized the instrument as “straightforward,” with a patient remarking that it asked “good, nitty gritty questions,” and clinicians describing it as “well-thought out” and “concise.” A clinician offered, “I think even adding a couple more questions [e.g., about employment, a patient’s support system, or religion] would be okay to make sure that the survey is comprehensively addressing the needs that many patients with cancer may be struggling [with].” To do so without increasing burden, caregivers and oncologists suggested condensing questions where possible and changing the lookback period to the past 30 days versus the past week. Patients recommended dividing items into clear sections. All participants suggested adding more detailed follow-up questions regarding housing stability (eg, renting/paying a mortgage, housing affordability assistance) and neighborhood safety. Patients and caregivers advised including questions about nutrition and supplements.

Patients and clinicians both stressed that care should be taken regarding how and where the instrument is completed. Patients offered that mode of administration (eg, in-person versus virtual) could affect their responses and encouraged translation into multiple languages. One patient cautioned that the instrument “assume[d] a reading level that’s relatively sophisticated,” although clinicians thought it was accessible to all educational backgrounds. Finally, a clinician noted that administering the instrument solely in clinic could be problematic: “When patients have socioeconomic burden…they don’t come to the clinic because they might not have transportation…it’d be like a sampling bias.” Overall, patients and caregivers advised proceeding with caution, noting patients could be embarrassed responding to some questions. One caregiver stressed, “I know my sister is proud, so if someone randomly…gave her this survey, she may not want to answer truthfully about what she is going through because we might not really know” if the responses will remain confidential or be addressed.

### Community resource referral document

For the content and layout of the community resource referral document (Table 4), participants liked that it provided comprehensive, clear information in one place, focused on key stressors, would be personalized to patients’ needs, and included information for a community navigator. A clinician elaborated, “[It’s] not just like, here are the resources and off you go… It’s also helpful to have someone who can personally guide you.” A patient summarized, “[T]he best part of it is the health system…showing some care about the other parts of my life…I haven’t heard that often.” Additional suggestions were to provide a contact person for each community organization, to regularly update the document, and to more widely publicize its availability.

Patients felt the document was well-organized, while clinicians liked that it could be used by both patients and clinicians. Patients and caregivers shared concerns that they might be too overwhelmed to initiate contact and follow up with community organizations. A caregiver explained, “On a normal day [my mother] is very overwhelmed…I cannot keep track of everything…so while we would prioritize using these services if we needed it, at that moment when it is given to us, it may be tough if there’s a lot of appointments or my mom’s feeling really stressed.”

A patient added that they may need encouragement to use the document:

Sometimes people need a little push to use these things. Sometimes they might be embarrassed to use these things. So perhaps, while people are…sitting and having chemotherapy, if somebody came in and said, “Do you need these services? Can I help you navigate any of these services?” That might be helpful. I think some people maybe don’t understand how available these things are.

## Discussion

Interest-holders were generally receptive to the integration of HRSC assessment and assistance with cancer care. Opinions varied as to what HRSCs were relevant to women’s experiences of ovarian cancer care and for which HRSCs cancer providers should screen. Whereas instruments like the Accountable Health Communities screening tool^23^ focus on housing, food, transportation, utilities, and interpersonal safety, our participants offered more expansive, nuanced interpretations of pertinent HRSCs. For instance, perhaps given the potentially life-limiting and gender-specific nature of advanced ovarian cancer, all participants recommended attending to spirituality/religion, mental health support, and interpersonal violence. Clinicians considered healthcare-adjacent concerns of insurance coverage, medication access, and disability to be HRSCs, while patients and caregivers emphasized neighborhood safety and nutrition guidance.

Opinions varied on which HRSCs are relevant for women with ovarian cancer, mirroring the lack of consensus around which HRSC screening tool to use in oncology and how best to refer to these patient-level social factors (eg, HRSCs versus health-related social risks).^29^^,^^30^^,^^31^^,^^32^ Standardized screening instruments have the benefits of facilitating cross-setting comparisons and prompting clinicians to discuss potentially sensitive topics like palliative/end-of-life care, interpersonal violence, and sexual function, which they might otherwise avoid; however, institutions might need to tailor HRSCs to their patient population, clinical setting, institutional resources, and personnel available to screen for and respond to HRSCs.

The way HRSC discussions occur in the clinical setting may be more important than where or with whom. Consistent with a scoping review,^33^ patients and caregivers were comfortable discussing HRSCs with nurses, social workers, and community advocates. Though ASCO de-emphasizes oncologists’ role in addressing HRSCs given their work burden,^14^ patients and caregivers endorsed discussing HRSCs with oncologists. Concordant with other studies noting patients’ concerns about privacy and the centrality of trust in discussing HRSCs, all participants acknowledged that patients could feel embarrassed sharing aspects of their social situation and that such discussions must occur with empathy, trust, and respect regardless of the clinician involved.^33–36^ For example, our patient participants endorsed discussion of interpersonal violence as a “real” topic. Participants in other studies have agreed, yet interpersonal violence was rarely reported, possibly indicating low incidence but potentially reflecting discomfort having the discussion despite its importance and impact.^37–39^

This emphasis on how HRSC discussions occur, rather than where they occur and with whom, has 4 downstream implications. First, whereas most research reports on single-timepoint screening,^29^ trust follows relationship-building.^40^ HRSC assessment and assistance may have to occur when rapport is established versus at the initial visit.^36^ Further, as clinician participants noted, discussions bear revisiting, as HRSCs can change over time. Second, although clinician participants endorsed viewing HRSC documentation in the EMR, patients prefer additional confidentiality measures with HRSC information in the EMR.^37–39^ Third, although research suggests patients often self-administer HRSC screening surveys via EMR patient portals,^29^ such methods may not foster empathy, engender understanding of why gathering such information is important or what follow-up will occur. As such, multi-modal HRSC assessment with in-person engagement may boost screening rates^41^ and support patients’ trust in the process and purpose of HRSC management within the clinical setting. Fourth, consistent with prior literature, our findings highlight that assessment without assistance can adversely affect trust.^32^^,^^36^ Even if patients do not request immediate assistance for unmet HRSCs, knowing that referral to resources is available, as one patient noted, can “show some care.”^35^ Awareness of patients’ unmet HRSCs can also enable cancer clinicians to adjust treatment in a patient-centered manner.

Beyond the positive reception garnered by the community resource referral document, participants’ feedback provided 2 additional insights. As found previously, participants had limited awareness of clinic-based resources and community-based organizations addressing HRSCs and were both eager to receive practical information and supportive of wider publicity of available resources.^35^^,^^42^ Although participants liked the potentially personalized nature of the document, oncology clinicians could consider universal delivery of community resource information at the initial visit with subsequent visits providing an opportunity for reminders of resource availability and discussion of additional needs.^35^^,^^43^ With no screening or assessment required, universal delivery can be a more cost-effective, sustainable, and stigma-reducing approach. Additionally, patients’ and caregivers’ potentially feeling overwhelmed by needing to contact community-based organizations themselves highlights the role of community navigators in increasing connections after referral.^36^^,^^44^ Technology-enabled navigation can augment^45^ but may not be able to replace community navigators in facilitating such connections, as ASCO recognizes.^14^

### Limitations

We note a few limitations to this study. First, although we interviewed only 4 to 5 individuals in each interest-holder group and all participants were from 1 institution, this purposive sample size was methodologically appropriate for this focused, preparatory work that prioritized clarity of participant perspectives over breadth or representativeness. Findings, however, may not be generalizable. Second, the RADaR approach, through its iterative data reduction, is designed to produce concise outputs that directly and precisely connect participants’ responses to the interview questions and domains. The resultant qualitative data may not be as rich or nuanced as other qualitative methods or approaches may produce. Third, some participants may have been uncomfortable talking about HRSCs, potentially limiting data, however, some participants broached sensitive issues unprompted. Fourth, because data were collected during the later stages of the COVID-19 pandemic, perspectives on HRSCs may reflect time-specific dynamics in social services, access to care, and resource availability. Nevertheless, this study represents an important step in understanding interest-holder perspectives on HRSCs in ovarian cancer care.

## Conclusion

To improve care experiences and quality of life and reduce socioeconomic-related outcome disparities for women with ovarian cancer, practical approaches to assess for and assist with HRSCs are needed. Creating workflows and marshaling resources for HRSC management is an iterative process. While reduction in acute healthcare utilization may offset social care cost,^43^ the more person-power required to deliver the social care, the harder it may be to sustain. We demonstrate interest-holders’ openness to discussing how to integrate HRSC management with clinical care and the value of triangulating their feedback to inform the practical considerations of who, what, when, where, why, and how HRSC assessment and assistance can be undertaken in oncology.

## Supplementary Material

oyag077_Supplementary_Data

## Data Availability

The data underlying this article will be shared on reasonable request to the corresponding author.
