# Supplementary material for: “Showing some care”: interest-holders’ perspectives on addressing health-related social conditions in ovarian cancer
Source: Oncologist. 2026 Mar 16;31(4):oyag077. doi: 10.1093/oncolo/oyag077 (PMC13035072; doi:10.1093/oncolo/oyag077)
Supplement: oyag077_Supplementary_Data [file oyag077_supplementary_data.docx]

**Supplemental File S1. Composite Health-Related Social Condition Screening Instrument Developed from Existing Validated Instruments including the Accountable Health Communities Tool, Medical Expenditure Panel Survey, and COST-FACIT Instrument**

**
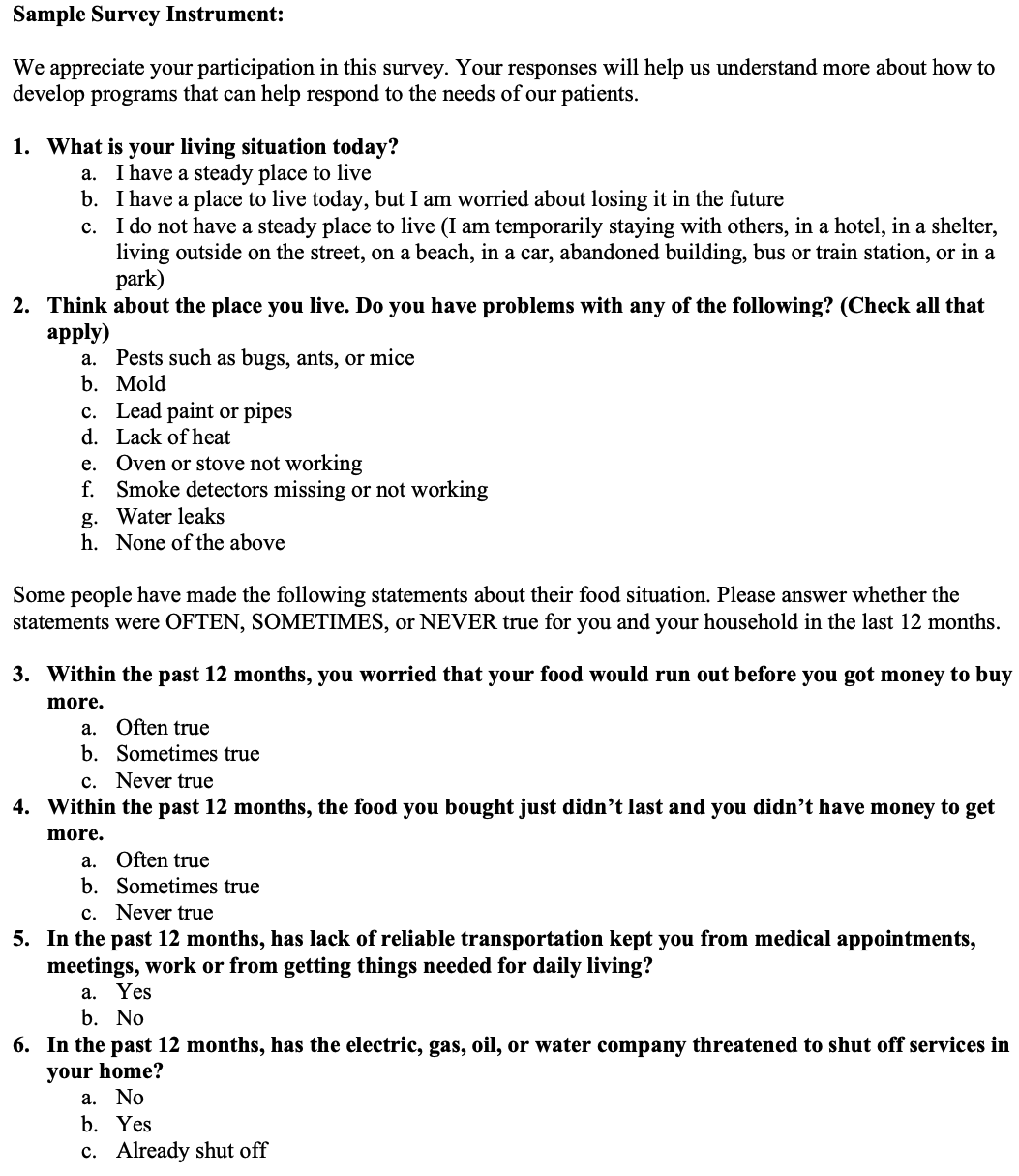
**

**
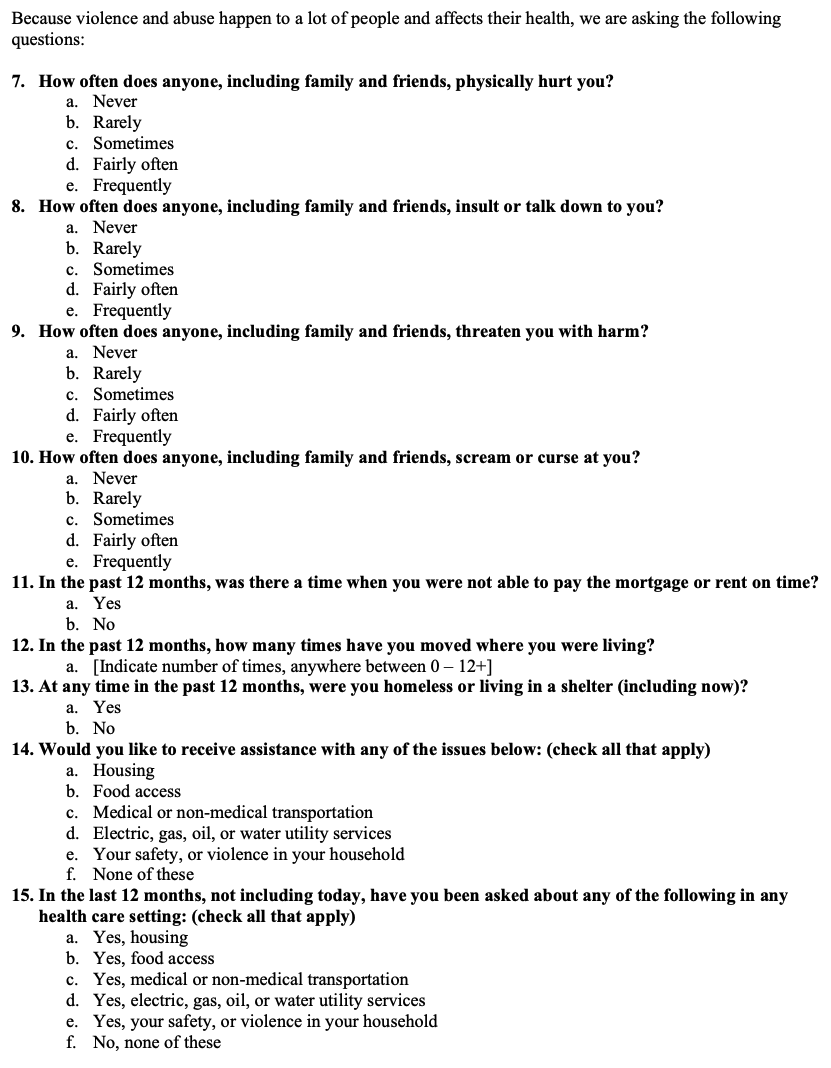
**

**
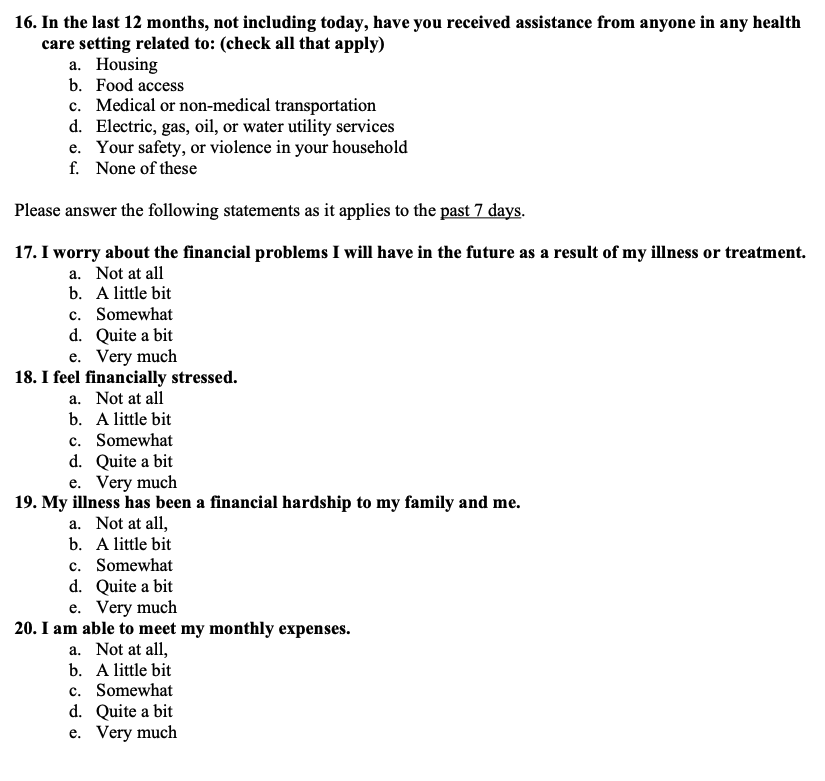
**

**Supplemental File S2. Sample Version of Community-Resource Referral Document**


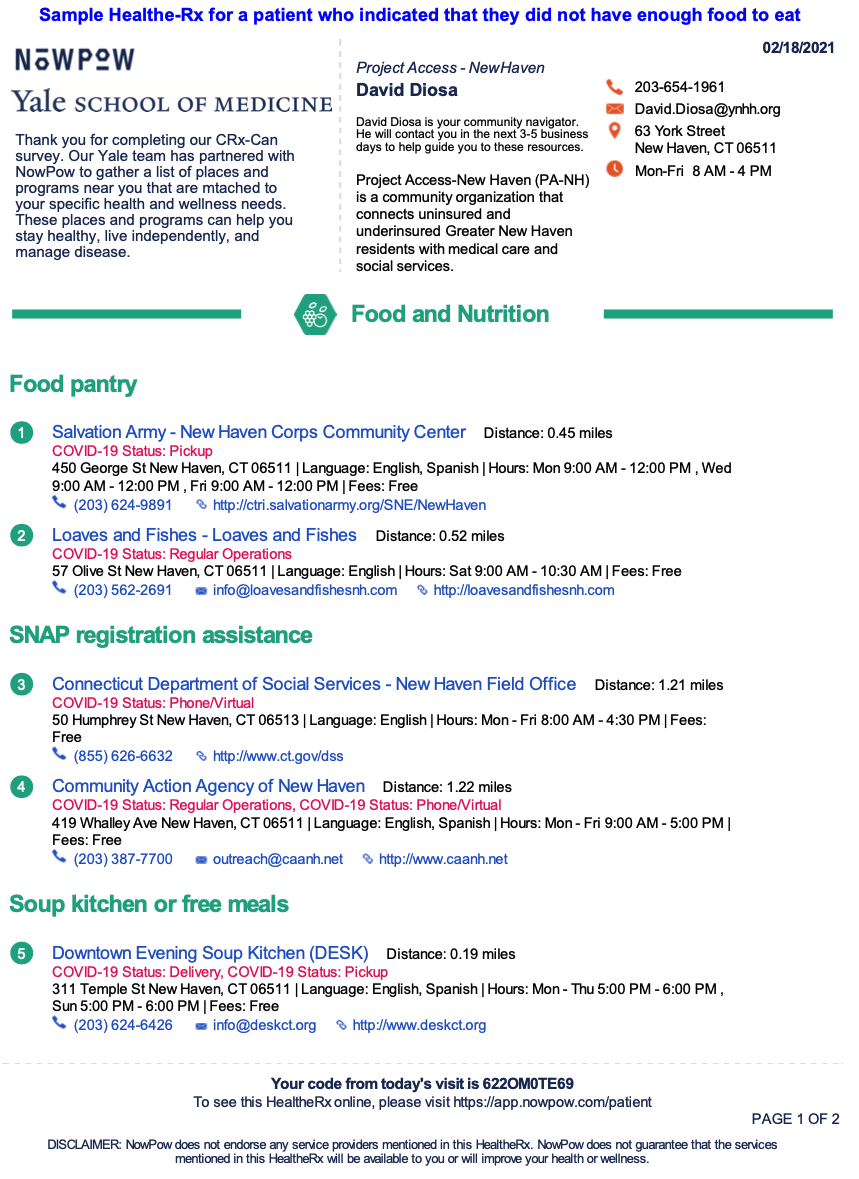


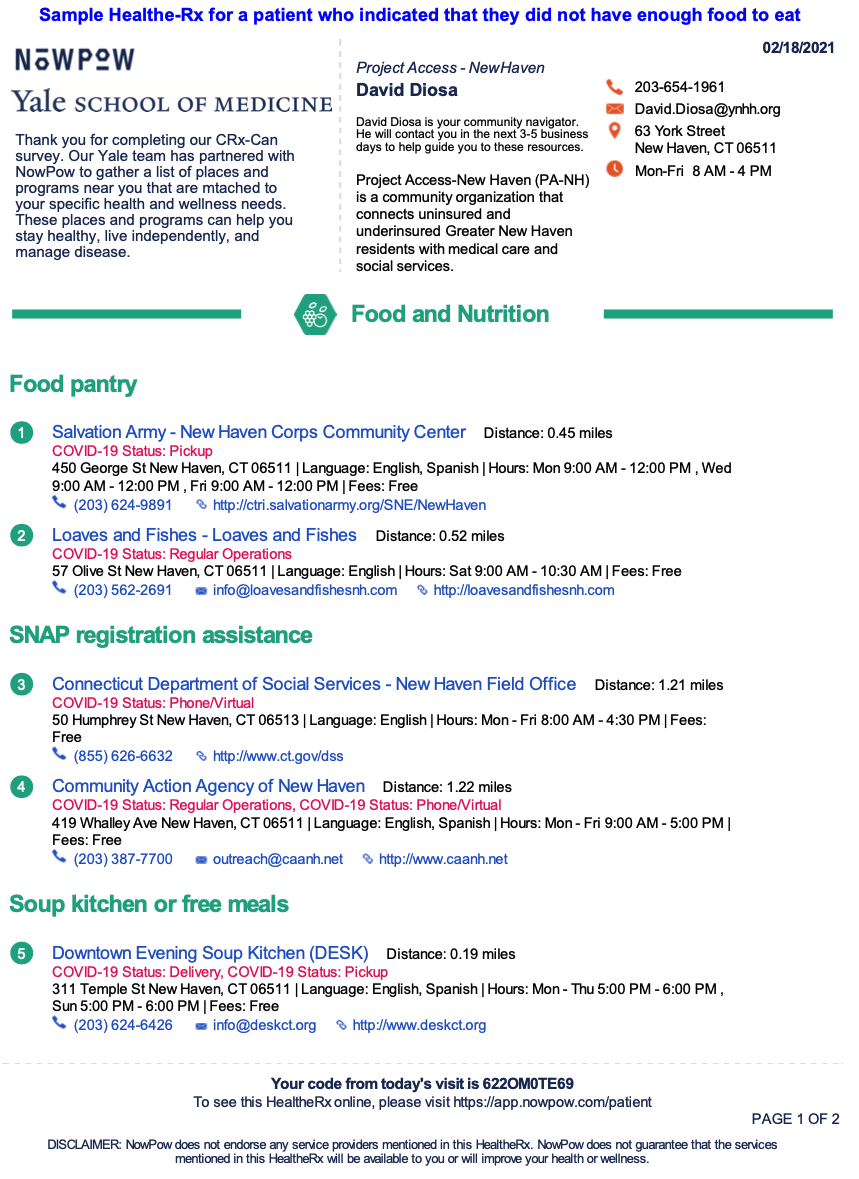


**Supplemental File S3. Patient and Family Caregiver Code Key**

1. **Additions to Diagram**
   1. Spiritual/prayer
   2. Psychological services
   3. Non-cancer medical treatment
   4. Employment
   5. Rename “wigs” as personal appearance
   6. No additions (ex. don’t go to community, good summary)
   7. Nutrition support
   8. Employment
2. **How feel about being asked about socioeconomic needs**

a. Yes, ask- II

b. No, don’t ask- 0

c. +/- - I

d. No response - II

1. **Which HCP would you want to talk about s-e needs with?**
   1. Social worker
   2. Resource counselor
   3. Nurse
   4. Surgeon
   5. Oncologist
   6. Health care professional who has had Stage III/IV ovarian cancer
   7. Community advocate/liaison (e.g. religious leader)
   8. Point person
   9. Caregiver differs from/defers to opinion from patient
   10. Primary care physician
2. **Survey instrument**
   1. Liked about survey instrument
      1. Interpersonal violence questions (e.g., C04 on abuse)
      2. Straightforward
      3. In-depth questions
      4. Questions about living situation
      5. Addresses “real” issues
      6. Questions about food situation
      7. Questions about transportation
      8. Questions about finances
   2. Disliked about survey instrument
      1. Social desirability effect
   3. Burdensome?
      1. Yes – caregiver - I
      2. No – patients - II; caregivers - II
      3. +/- - caregiver - I
      4. No response – patients – III; caregiver -
   4. Changes/Suggestions for survey instrument
3. Nutrition/Supplements
4. Prognosis
5. Spiritual issues
6. Neighborhood safety
7. Health insurance/employment
8. Caregiving responsibilities
9. Survey administration (e.g., in-person/Zoom, in all languages)
10. Inclusion of rural health issues
11. Break items into clear sections
12. Check reading level
13. Mental health support
14. More detailed questions (e.g., for housing – housing stability, rent/own, mortgage)
15. Change lookback period to last month vs. last 7 days (C07)
16. Combine questions where possible (C07 – “some of the questions are kind of asking the same thing”)
17. Consider adding free response (C07)
18. **Health eRx**
    1. Liked about Health eRx
       1. Meets a need
       2. Good idea/concept overall
       3. Offers an alternative to existing supports
       4. Info in one place
       5. Holistic
       6. Clarity/well-organized
       7. Worth the effort
       8. Color-coded
       9. Presence of primary contact person
       10. Personalized to patients’ needs
    2. Disliked about Health eRx
       1. Burdensome/too overwhelmed to act upon
    3. Changes/Suggestions for Health eRx
       1. People may need a push to use HealtheRx
       2. Availability/nature of HealtheRx should be publicized
       3. Recruit community advocates (e.g. church leader)
       4. Clearer/specific contact information
       5. Make more concise/offer as a table (C04)
       6. Clarify any out-of-pocket costs for services (C07)
    4. NowPow relevance
19. Yes- patients: 0; caregiver: I
20. No- patients: II; caregiver: I
21. +/-- patients: II
22. No response – patients: I; caregiver: II

^a^Code numbers do not correspond to interview question numbers.

**Supplemental File S4. Oncology Clinician Code Key^a^**

1. **Additions to Diagram**
   1. Medication access
   2. Insurance
   3. Religion
2. **How feel about asking socioeconomic needs**

a. Yes, ask - III

b. No, don’t ask-

c. +/- - II

1. **Frequency of asking about socioeconomic needs**
   1. Quarterly - I
   2. At first visit and anytime thereafter - I
   3. Based on chemotherapy visits – II
   4. Every couple of months- I
2. **View socio-economic needs in medical record?**
   1. Yes- III
   2. No-
   3. +/- -- II
3. **Survey instrument**
   1. Liked about survey instrument
4. Brief/not overwhelming
5. Straightforward / well-thought out
6. Items get at important information
7. Inclusion of financial needs
8. Accessible to all educational backgrounds
9. Inclusion of family and friends
10. Inclusion of interpersonal violence
    1. Disliked about survey instrument
    2. Burdensome?
       1. Yes - I
       2. No – III
       3. +/- - I
    3. Changes/Suggestions for survey instrument
11. Hoarding
12. Ask about stairs
13. Places to get furniture
14. Prescription assistance
15. Change in housing
16. Short/long term disability
17. Insurance (Social Security/Coverage)
18. House cleaning
19. Senior center programing
20. Location for completion
21. Look back period
22. Condense similar questions
23. **Health eRx**
    1. Liked about Health eRx
24. Direct contact with an external person
25. Focus on key stressors
26. Personalized
27. Important info (websites, phone numbers, etc.)
28. Quick to access
29. Holistic
30. Well written/comprehensive
31. Easy to follow
32. Includes COVID content
33. Layout
34. Co-use for patients and providers
    1. Disliked about Health eRx
    2. Changes/Suggestions for Health eRx
35. Add new organization
36. More examples of resources
    1. NowPow relevance
37. Yes-II
38. No-III

^a^Code numbers do not correspond to interview question numbers.
